# Evidence based healthcare: encouraging the adoption of a new philosophy of care

**DOI:** 10.1590/S1678-77572010000400001

**Published:** 2010

**Authors:** Z Fedorowicz, JT Newton

**Affiliations:** Director, Bahrain Branch UKCC The Cochrane Collaboration; Professor of Psychology as Applied to Dentistry, King’s College London Dental Institute

A sound knowledge of the effectiveness of healthcare interventions is not only a requirement but an ethical imperative for all clinicians. Appeals for the improved assessment of effectiveness in healthcare interventions have an extensive history. The most recent of these has led to the growth of the evidence-based healthcare movement, generally believed to have originated in the late 70’s with the work of White, Cochrane and Illich, the unreservedly recognised giants of clinical epidemiology. The World Health Organisation (WHO) added to the momentum through the timely release of a statement at the Alma-Ata Conference^6^ (1979) which was an international clarion call for best evidence to be used in guiding healthcare decision making. At this conference the Director General of the WHO, Halfdan Mahler, succeeded in coalescing the often disparate views and agendas of the representatives of 134 governments and crafted a statement which may now be considered prophetic for its time. In this statement the delegates unanimously agreed that, "the time has come for all levels of the health system to review critically their methods, techniques equipment and drugs, with the aim of only using those technologies that have really proved their worth and can be afforded"^6,16^. A decade later Gordon Guyatt^8^ (1991) coined the phrase "evidence-based medicine" and the advocates of evidence-based medicine proclaimed a shift to a "new paradigm", in which healthcare decisions should be based on the best available evidence obtained through the use of robust research methods^7^.

Sackett and his colleagues define Evidence-based medicine (EBM) as, "the conscientious, explicit and judicious use of current best evidence in making decisions about the care of individual patients"^14^. The inadequacy of this definition, by apparently excluding the patient from the decision process, motivated its subsequent revision by Sackett to highlight the contemporary view of the patient as a partner in the concept and it is now defined as, "the integration of best research evidence with clinical expertise and patients values"^13^. The recrafting of this definition appears to have "strengthened" the contribution of clinicians’ individual skills, conceivably to allay their fears of a "prescriptive" slant to the concept.

The discrepancies which occur with a reliance on expert knowledge alone have been highlighted by Antman, et al.^2^ (1992) who examined the accumulated evidence, over the period 1960-1990, on the effectiveness of anti-arrhythmic and other medications, to reduce the risk of heart attack. They then compared the "current best evidence" to what interventions the "experts" were recommending. Their study found major discrepancies between the accumulating evidence and the experts’ recommendations. In most instances where studies showed treatments to be effective, experts’ recommendations lagged several years behind the evidence. In summary they noted that some experts "have not yet mentioned effective therapies, while others continue to recommend those that are ineffective or possibly harmful"^2^.

Evidence-based healthcare then seeks to support clinicians in choosing healthcare interventions through the identification of high quality research evidence as a basis for decision making. What impact to date has this had on the specialties of oral surgery, oral medicine, oral pathology, oral radiology and endodontics? Taking one of the leading journals that cover these specialties as an example: between January 2001 and July 2005 Oral Surgery Oral Medicine Oral Pathology Oral Radiology & Endodontics published 3 articles^3,9,13^ claiming to be systematic reviews out of 1,184 articles published in that same time period. However none of these were registered with the Cochrane Collaboration, and of the 5 "systematic reviews" none were reviews of randomized controlled trials or controlled clinical trials alone.

Dental specialty journals are not unusual in this slow adoption of evidence-based dentistry. Despite a burgeoning interest in the conduct of systematic reviews and the production of clinical guidelines, interest and adoption of evidence based healthcare has generally been slow amongst clinicians and educators despite its early connections with medical education^5^. Initially it would appear that the evidence based medicine movement assumed that the existence of high quality evidence, summarized in systematic reviews and published in accessible formats would be of itself sufficient to induce change in practice. However it has been recognized that such "passive" means of dissemination are insufficient to produce significant change in behaviour^4^. In 1994 the Cochrane Effective Practice and Organisation of Care (EPOC) group was established to identify methods which could be adopted to improve professional practice and the delivery of effective health services^1,11^. Of course, in the tradition of evidence-based medicine, the EPOC group achieves this aim through undertaking systematic reviews of the effectiveness of methods to improve practice. There is a growing recognition that these methods should include organizational, financial and regulatory interventions, rather than simply focusing on education.

McGlone, et al.^10^ (2001) summarise the barriers to the uptake of evidence-based dentistry by practitioners as: knowledge and attitude of the practitioner; patient factors; practice environment; educational environment; the wider health system, and the social environment. McGlone and colleagues argue that the culture of a society will determine patients’ expectations of medical and dental encounters, for example many patients attending a doctor or dentist in Western society will have expectations of prescription, either of drug or other treatment leading to for example the inappropriate prescribing of antibiotics^12^. Such cultural influences on the adoption of evidence based healthcare are likely to vary across countries. The culture of healthcare practice will be in part determined by the learning experiences of healthcare professionals during training. The concepts and methods of evidence-based healthcare should form the foundation of the teaching and learning of healthcare professionals.

A health system which includes remuneration for treatment will encourage a practitioner to prescribe treatment regardless of effectiveness (particularly if the practitioner is not aware of evidence suggesting the intervention to be ineffective or harmful), and suggests that healthcare (and remuneration) should be organized to encourage the adoption of care based on the best available evidence.

Having identified the barriers to the adoption of evidence-based healthcare, what effective techniques can be adopted to promote a change in the behaviour of healthcare professionals. From a systematic review of 18 systematic reviews Bero, et al.^4^ (1998) were able to identify interventions which have consistently been found to be effective in promoting behavioural change in healthcare professionals: educational interventions were effective if they included an element of outreach with education taking place in the workplace, and if they were interactive (that is they include discussion or practice of skills); reminders (either manual or computerized systems for reminding healthcare professionals); interventions which involved combinations of methods such as reminders, audit and feedback, involvement of professionals in devising guidelines. Educational materials and didactic educational meetings were consistently found to have little or no effect on uptake of evidence-based care.

If evidence-based healthcare is to occupy a central role, as it should, in the decision making process of clinicians then it is not sufficient for research to continue to provide the best evidence and hope that it is taken up by clinicians, active processes of dissemination should be adopted including the introduction of structural changes (such as modifications to systems of remuneration) that encourage the practice of evidence based healthcare. Perhaps the most appropriate technique for ensuring that evidence based dentistry is adopted by practitioners is to encourage researchers to conduct and publish systematic reviews. A recent Lancet editorial states that in future clinical trials will not be accepted for publication in that journal unless there it is clear from a systematic review of the literature that indicates the necessity for a new trial^17^. Such an approach will avoid the unnecessary repetition of trials where a clear clinical effect (or lack of effect) has been established. We recommend such a policy to the editorial boards of all dental journals.
